# Post-pancreatectomy clip migration: a two-stage management approach with pancreatography and pancreatoscopy

**DOI:** 10.1055/a-2819-1367

**Published:** 2026-03-11

**Authors:** Yuya Tanaka, Reika Matsushita, Yoki Endo, Katsushi Suenaga, Shimpei Takagi, Taihei Soma, Yoshifumi Ohnishi

**Affiliations:** 1 26357 Department of Surgery, NHO Shizuoka Medical Center, Shimizu, Japan; 2 4615 Imperial College London, London, United Kingdom; 3 20084 US Naval Hospital Yokosuka, FPO AP, United States; 4 26357 Department of Gastroenterology, NHO Shizuoka Medical Center, Shimizu, Japan

Clip migration is a rare complication occurring mostly after cholecystectomy. Upfront retrieval with endoscopic retrograde cholangiopancreatography (ERCP) is the standard treatment for post-cholecystectomy clip migration (PCCM 1 ). However, performing invasive procedures during pancreatitis is associated with increased mortality. We report a safely managed post-pancreatectomy clip migration (PPCM) into the main pancreatic duct (MPD).

**Fig. 1:**
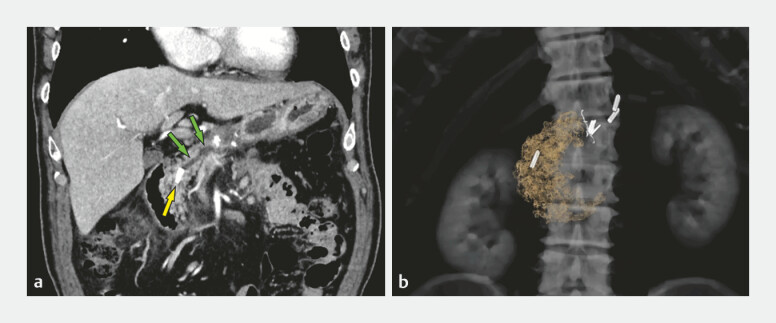
Post-pancreatectomy clip migration. **a** Surveillance CT reveals an incidental finding of clip migration (yellow arrow) into the MPD causing distal dilation (6 mm, green arrows), with pseudocyst formation and mild obstructive pancreatitis. **b** 3D reconstructed CT reveals the double-shank form of the clip. CT, computed tomography; MPD, main pancreatic duct.

A 77-year-old man was recalled to the hospital 3 months after undergoing uncomplicated laparoscopic distal pancreatectomy for non-invasive intraductal papillary mucinous carcinoma of the pancreatic body because surveillance computed tomography revealed a clip lodged in the MPD causing obstructive pancreatitis ( Fig. 1 ). Although he was asymptomatic with non-contributory physical examination, serum amylase (125 IU/L) and C-reactive protein (17.8 mg/dL) levels were elevated.

**Fig. 2:**
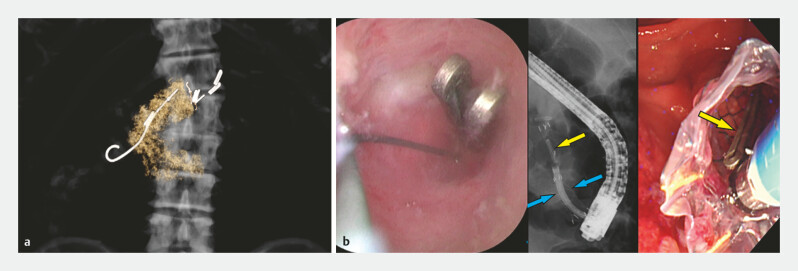
A two-stage management approach. **a** Stage 1: sphincterotomy and pancreatic drainage tube placement under ERCP to resolve the obstruction and allow for spontaneous clip passage (results shown in 3D CT). **b** Stage 2: clip retrieval (yellow arrow) under pancreatoscopy using a snare (SpyGlass Retrieval Snare 9 mm × 286 cm; Boston Scientific, MA, USA), with additional fully covered stenting (HANAROSTENT Biliary [CCC] 8 mm × 50 mm; M.I.Tech, Pyeongtaek, South Korea, blue arrows) to dilate the MPD. CT, computed tomography; ERCP, endoscopic retrograde cholangiopancreatography; MPD, main pancreatic duct.

**Fig. 3:**
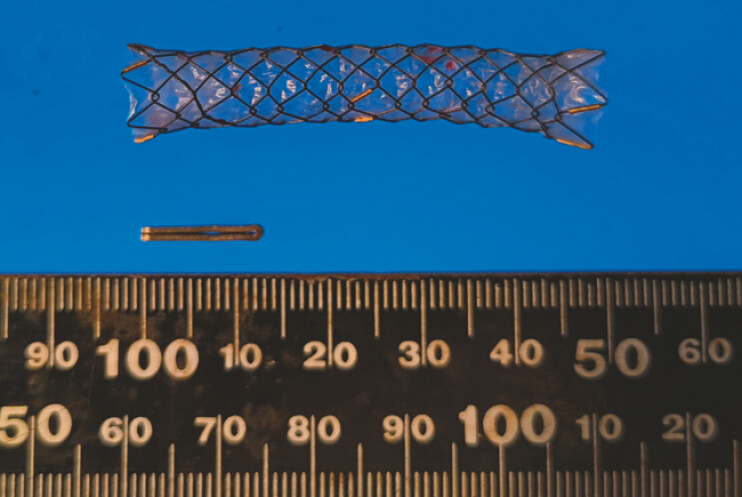
The retrieved 12 mm clip (DS Titanium Ligation Clips; Aesculap, Tokyo, Japan) and the covered stent.

A two-stage approach was taken to mitigate the risk of acute-on-chronic pancreatitis ( Fig. 2 ): first, ERCP was performed with sphincterotomy and pancreatic stent placement after which the patient was discharged; second, elective pancreatoscopy (SpyScope DS II; Boston Scientific, MA, USA) performed ( Video 1 ). Initial retrieval attempts under fluoroscopy failed due to the lack of direct clip visualisation. A fully covered metal stent was utilised to pass the pancreatoscope into the MPD, and allow the clip to be pulled into and out with the stent ( Fig. 3 ). The postprocedural course was uneventful.

Stage 2 of the two-stage management approach to post-pancreatectomy clip migration.Video 1

**Fig. 4:**
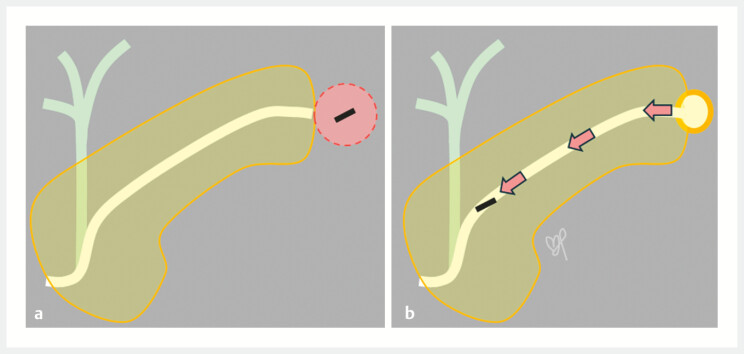
The postulated mechanism of MPD clip migration. **a** Subclinical pancreatic duct leak lyses the surrounding tissue freeing the clip, which was applied to branches of CIPV for haemostasis. **b** The clip is walled-off within the pseudocyst eventually draining into the MPD carried by the pancreatic fluid. CIPV, centro-inferior pancreatic vein; MPD, main pancreatic duct.

One case of PPCM has been reported previously into the stomach through a post-endoscopic ultrasound-guided-cyst drainage scar 2 . We extrapolate that fistula formation, a postulated mechanism also for PCCM, is applicable to the present case ( Fig. 4 ). Cholangioscopy is necessary for complex PCCM with unsuccessful ERCP 3 or with clips embedded in the fibrotic tissue 4 ; likewise, visualisation of the clip was essential for the meticulous procedure within the limited working space of the MPD.

We highlight this first description of MPD-PPCM, where the two-stage approach with ERCP and pancreatoscopy may be a safe and feasible option.

Endoscopy_UCTN_Code_TTT_1AR_2AG Endoscopy_UCTN_Code_TTT_1AR_2AI
